# DFT-Based Design and Characterization of Organic Chromophores Based on Symmetric Thio-Bridge Quinoxaline Push–Pull (STQ-PP) for Solar Cells

**DOI:** 10.3390/molecules31060927

**Published:** 2026-03-11

**Authors:** Edwin Rivera, Alex Garavis, Juan Garcia, Oriana Avila, Ruben Fonseca

**Affiliations:** Optical Spectroscopy and Laser Group (GEL), Popular University of Cesar, Valledupar 200001, Colombia; agaravis@unicesar.edu.co (A.G.); jmgarciazuleta@unicesar.edu.co (J.G.); orianaavila@unicesar.edu.co (O.A.); rubenfonseca@unicesar.edu.co (R.F.)

**Keywords:** organic solar cells, chromophores, density functional theory, thio-bridge quinoxaline

## Abstract

Organic solar cells require molecular materials with broad absorption and proper energy-level alignment to maximize photon harvesting and charge transport; in this context, this work focuses on the computational design and characterization of π-conjugated push–pull chromophores, providing an integrated evaluation of their electronic, thermodynamic, and optoelectronic properties for photovoltaic applications. The chromophores were optimized using DFT/ b3lyp/6-31g+(d,p) in Gaussian16, incorporating solvation effects through the CPCM model. Electronic, thermodynamic, and optical properties were investigated using DFT and TD-DFT/CAM-B3LYP/6-311+G(d,p), including the calculation of absorption and emission spectra, first hyperpolarizability, and two-photon absorption. The STQ-PP chromophores exhibit differentiated optoelectronic responses, with DTTQ-DPP-1 showing an energy gap of 0.82–0.86 eV, stabilized LUMO levels between −2.50 and −2.61 eV, high electronic polarizability, and optical absorption extended beyond 800 nm, favoring the harvesting of low-energy photons, whereas DTTQ-DPP displays a gap close to 2.70 eV and absorption predominantly localized in the UV region, associated with potentially inferior photovoltaic performance. Compared with commercial donor materials, DTTQ-DPP-1 exhibits a red-shifted absorption into the NIR and a smaller gap, indicating enhanced low-energy photon capture; its structural stability and increased rigidity further support its photovoltaic viability.

## 1. Introduction

Research on organic solar cells (OSCs) has advanced significantly over recent decades, driven by the pursuit of lightweight, flexible, and low-cost photovoltaic technologies based on molecular materials with tunable electronic and optical properties [1,2,3,4,5]. In this context, the rational design of π-conjugated organic chromophores with donor–acceptor (push–pull) architectures has become a key strategy to optimize light harvesting, exciton separation, and charge transport, which are decisive factors for the photovoltaic performance of these devices [6,7,8].

In commercial and widely studied organic materials for photovoltaic applications, such as conjugated donor polymers and fullerene-type acceptors (for example, PCBM-based systems), the HOMO energy levels are typically located in the range of −5.8 to −5.5 eV, while the LUMO levels are found around −3.9 to −3.8 eV, ensuring efficient charge separation and adequate electron transport [9,10,11]. Consistently, these materials exhibit an optical band gap (E_9_) between 1.6 and 1.8 eV, which is considered optimal for efficient absorption in the visible region of the solar spectrum [12]. From an optical standpoint, the active layers of commercial organic solar cells display broad absorption covering approximately the 400–800 nm range, enabling power conversion efficiencies on the order of 10–15%, which serve as a direct benchmark for the design and evaluation of new organic chromophores for photovoltaic applications [13,14].

Within this framework, molecular optoelectronic properties, such as the alignment of frontier molecular orbitals (HOMO and LUMO), the magnitude of the electronic band gap, polarizability, and the degree of intramolecular charge transfer, play a central role in photovoltaic efficiency [15,16]. In particular, the ability to extend absorption beyond the visible region into the near-infrared is especially attractive for harvesting lower-energy photons, a portion of the solar spectrum that remains partially underutilized by many conventional organic materials [17,18].

**Figure 1:**
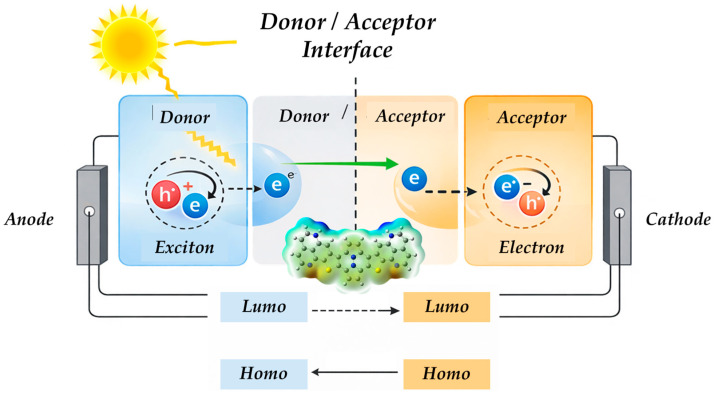
Schematic representation of the operating principle of an organic solar cell (OSC), illustrating light absorption in the donor material, exciton generation and diffusion toward the donor/acceptor interface, charge separation driven by HOMO/LUMO energy level alignment, and subsequent electron and hole transport toward the cathode and anode, respectively.

Figure 1 presents a schematic representation of the operating principle of an organic solar cell (OSC), clearly distinguishing the donor material, the donor/acceptor interface, and the acceptor material, which are connected to the anode and cathode, respectively. Incident solar radiation is absorbed by the donor, generating an exciton (a bound electron–hole pair) that diffuses toward the interface, where the energetic alignment between the HOMO and LUMO levels promotes charge separation: the electron is transferred to the acceptor LUMO while the hole remains in the donor HOMO. Subsequently, the electron is transported through the acceptor toward the cathode, and the hole migrates through the donor toward the anode, producing electrical current in the external circuit; the lower part of the scheme illustrates the corresponding HOMO and LUMO energy levels, highlighting the energetic driving force responsible for efficient charge separation and transport.

In a complementary manner, nonlinear optical (NLO) properties have emerged as fundamental descriptors of the electronic quality of advanced organic chromophores [19,20]. Quantities such as the first hyperpolarizability and two-photon absorption (TPA) are directly related to electronic polarizability, π-delocalization, and the strength of intramolecular charge transfer—features that are also essential for efficient optical absorption and favorable charge transport in photovoltaic devices [21]. Although nonlinear processes do not constitute the operative mechanism under standard solar illumination conditions, their analysis provides valuable intrinsic information on the nature of low-lying excited states, electronic coupling, and the response of the system to intense electric fields, which are relevant for the design of advanced optoelectronic materials and next-generation photovoltaic architectures [22].

In this regard, chromophores based on heteroaromatic acceptor cores, combined with π-conjugated spacers and suitable donor fragments, have proven to be highly versatile platforms for precisely tuning both linear and nonlinear optical properties [23,24]. The incorporation of heteroatoms such as nitrogen and sulfur contributes to the stabilization of electronic energy levels, enhances π-delocalization, and promotes excited states with pronounced charge-transfer character, resulting in more intense absorption shifted toward longer wavelengths, as well as an amplified nonlinear optical response [25].

In recent years, push–pull organic systems have been extensively investigated due to their ability to modulate intramolecular charge transfer (ICT), reduce the electronic band gap, and extend absorption toward the visible and near-infrared regions—properties that are crucial for organic solar cells and optoelectronic devices [26]. Several studies have demonstrated that quinoxaline-based cores function as efficient electron-accepting units, enhancing π-conjugation and structural stability, while thiophene bridges increase molecular coplanarity and strengthen donor–acceptor electronic coupling, promoting effective spatial separation between HOMO and LUMO levels [27].

Symmetric A–D–A and D–A–D architectures incorporating quinoxaline units have shown improved visible absorption and enhanced nonlinear optical responses [28]. However, a systematic understanding of how subtle modifications in peripheral donor units simultaneously influence ICT strength, NIR spectral extension, and overall electronic responsiveness remains limited [29]. In this context, DTTQ-DPP and DTTQ-DPP-1 were designed as symmetric thio-bridge quinoxaline push–pull (STQ-PP) model systems to investigate the structure–property relationship between molecular architecture, electronic polarization, and linear/nonlinear optical performance, contributing to the rational design of low-band-gap organic chromophores.

Theoretical approaches based on density functional theory (DFT) and its time-dependent extension (TD-DFT) have become indispensable tools for the study of these systems, as they allow direct correlations to be established between molecular structure and electronic, thermodynamic, and optical properties relevant to solar applications [30,31,32,33,34,35]. Through these methods, it is possible to systematically evaluate energetic stability, intrinsic chemical reactivity, optical absorption and emission, as well as parameters associated with nonlinear optical response, providing solid criteria for the selection and optimization of new chromophore candidates for organic solar cells [36,37].

In this work, a detailed theoretical study of π-conjugated organic chromophores with a push–pull architecture is presented, aimed at comprehensively analyzing their optoelectronic and nonlinear optical properties relevant to photovoltaic applications, including extended absorption in the visible–near-infrared region, frontier orbital energy alignment, thermodynamic stability, the nature of excited states, and nonlinear response parameters. Through this approach, molecular design criteria are proposed to contribute to the development of more efficient active materials for the next generation of organic solar cells.

## 2. Results

In this section, the design and characterization of organic chromophores based on symmetric thio-bridge quinoxaline push–pull (STQ-PP) systems for solar cells are presented. The study employs density functional theory (DFT) computational algorithms, and the characterization is focused on electronic properties, intramolecular charge transfer (ICT), optical properties of one-photon and two-photon absorption, and first hyperpolarizability.

### 2.1. Design

**Figure 2:**
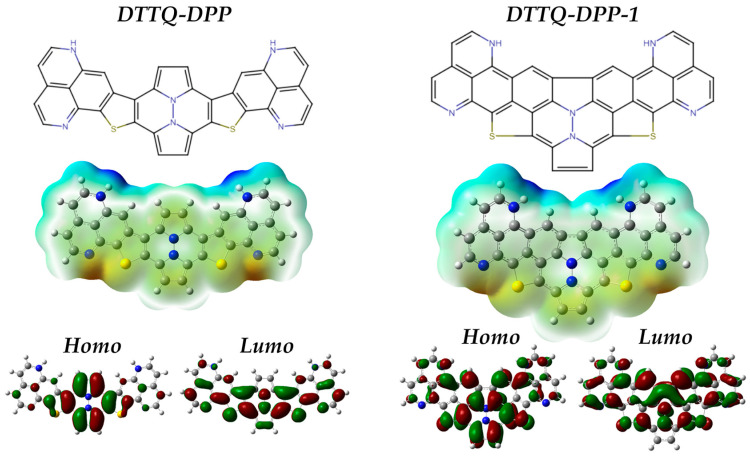
Structure, electron density distribution and HOMO–LUMO orbitals.

In Figure 2, the chromophores DTTQ-DPP (4,7-bis[5-(diazanaftalenyl)thiophen-2-yl]quinoxaline) and DTTQ-DPP-1 (4,7-bis[5-(triazanaphthalenyl)thiophen-2-yl]quinoxaline) were rationally designed as conjugated push–pull organic systems aimed at applications in organic solar cells, following a common molecular strategy that combines structural symmetry, extended conjugation, and efficient intramolecular charge transfer (ICT) [29]. Both compounds exhibit a well-defined D–A–D (Donor–Acceptor–Donor) molecular architecture, in which the central quinoxaline core acts as the electron-accepting unit and is symmetrically flanked by thiophene-based aromatic donor fragments [38]. These units are connected through thioconjugated bridges that promote electronic delocalization and ensure effective electronic communication along the molecular backbone.

From an atomic composition perspective, these chromophores are primarily composed of carbon (C) atoms, which build the aromatic framework and the extended π-system responsible for light absorption [39]; hydrogen (H) atoms, which stabilize the molecular skeleton without significantly perturbing the electronic structure [40]; nitrogen (N) atoms, located within the quinoxaline core and terminal heteroaromatic units, contributing strong electron-withdrawing character, stabilizing the LUMO energy levels and promoting ICT [41]; and sulfur (S) atoms, incorporated into the thio-bridges, which enhance molecular polarizability and facilitate electronic coupling between donor and acceptor segments due to their diffuse valence orbitals [42].

The conjugated C=C and aromatic C–C bonds enable continuous electron delocalization across the molecular framework, which is essential for strong optical absorption [25,43]. C–N bonds reinforce the electron-accepting nature of the central core and modulate charge distribution, while C–S bonds act as efficient charge-transfer pathways, strengthening donor–acceptor interactions and contributing to gap narrowing [25]. Together, these bonding features favor a quasi-coplanar molecular geometry, reducing torsional disorder and maximizing orbital overlap.

The structural difference between DTTQ-DPP and DTTQ-DPP-1 lies in subtle modifications of the peripheral donor units, which modulate the ground-state electronic polarization and, consequently, precondition the strength of intramolecular charge transfer (ICT) upon excitation. While ground-state descriptors alone do not define excited-state behavior, the observed electronic asymmetry provides a structural framework that facilitates ICT-dominated excited states, as reflected in the absorption features, large Stokes shifts, and nonlinear optical responses discussed below. In both cases, the molecular framework is designed to promote broad visible absorption, controlled HOMO–LUMO gaps, and enhanced electronic responsiveness, consistent with the push–pull architecture.

Compared with widely used organic photovoltaic materials—such as donor polymers P3HT [44], PTB7 [45], and PM6 [46], as well as common acceptors including PCBM and non-fullerene derivatives like Y6—the proposed chromophores offer potential advantages associated with their well-defined molecular structures, high conjugation efficiency, and tunable electronic properties [47,48]. Their rigid and symmetric architectures may lead to reduced energetic losses, improved level alignment, and enhanced optical absorption control. Moreover, the molecular nature of DTTQ-DPP and DTTQ-DPP-1 may provide improved structural reproducibility and design flexibility relative to polymer-based systems, which are relevant aspects for the development of next-generation organic photovoltaic materials.

Gas-phase and polar continuum calculations are used comparatively to assess intrinsic molecular properties and environmental sensitivity. While the gas phase reflects unperturbed electronic behavior, the polar medium provides an estimate of electrostatic stabilization, particularly relevant for intramolecular charge-transfer states, without explicitly modeling OPV devices.

### 2.2. Electron Density Distribution and HOMO–LUMO Orbitals

Figure 2 presents a comparative analysis of the molecular electrostatic potential (MEP) maps and the spatial distribution of the frontier molecular orbitals (HOMO and LUMO) of the DTTQ-DPP and DTTQ-DPP-1 chromophores.

In the upper panel, the MEP maps are shown, where regions depicted in red correspond to negative electrostatic potential associated with electron-rich domains, mainly localized around the nitrogen atoms of the quinoxaline core and, to a lesser extent, near the sulfur atoms of the thio bridges. In contrast, blue regions indicate positive electrostatic potential, characteristic of electron-poor areas associated with the peripheral donor fragments. Although the molecules are structurally designed as D–A–D systems, the MEP and CDD results indicate an effective A–D–A-like electronic polarization due to intramolecular charge redistribution. This reflects a difference between structural topology and electronic behavior.

In the lower panel, the HOMO and LUMO orbitals are illustrated. The HOMO is predominantly delocalized over the aromatic donor fragments and the thio bridges. In contrast, the LUMO is mainly localized on the quinoxaline acceptor core but also exhibits significant delocalization toward the peripheral acceptor fragments along the conjugated backbone. This extended spatial distribution of the LUMO, together with the HOMO localization on donor units, is fully consistent with a push–pull electronic structure and supports an efficient intramolecular charge-transfer mechanism upon optical excitation.

### 2.3. Electronic Descriptors and Intrinsic Chemical Reactivity

Table 1 summarizes the main electronic descriptors and intrinsic chemical reactivity parameters of the DTTQ-DPP and DTTQ-DPP-1 chromophores calculated in the gas phase and in methanol, allowing the analysis of both the structural effect and the influence of a polar environment on their electronic behavior.

The calculated total energies show that DTTQ-DPP exhibits values of −66,129.13 eV in the gas phase and −66,129.79 eV in methanol, whereas DTTQ-DPP-1 presents total energies of −68,167.68 eV in the gas phase and −68,168.36 eV in methanol. These values are reported as computational references and are not interpreted in terms of absolute stability between systems of different composition. Nevertheless, when each molecule is compared with itself, the inclusion of the solvent leads to an additional stabilization of the ground state in both cases, with a decrease of approximately 0.66 eV upon going from the gas phase to methanol.

Regarding the frontier molecular orbitals, DTTQ-DPP exhibits HOMO energies of −4.31 eV (gas) and −4.58 eV (methanol), whereas in DTTQ-DPP-1, these values shift to −3.32 eV and −3.47 eV, respectively, indicating an upward shift in the HOMO level associated with a stronger contribution of the donor units and a more extended conjugation pathway. Meanwhile, the LUMO energies decrease from −1.60 eV (gas) and −1.87 eV (methanol) in DTTQ-DPP to −2.50 eV (gas) and −2.61 eV (methanol) in DTTQ-DPP-1, reflecting a more effective stabilization of the acceptor level induced by the A–D–A architecture and enhanced intramolecular electronic coupling. This simultaneous HOMO elevation and LUMO stabilization explains the significant reduction in the electronic gap in DTTQ-DPP-1 and the emergence of low-energy transitions with pronounced intramolecular charge-transfer (ICT) character.

However, these LUMO values remain shallower than those of classical electron acceptors commonly employed in OPV devices (e.g., fullerene derivatives with LUMO energies around −4.0 eV). Therefore, DTTQ-DPP-1 should be regarded as a chromophore with relatively improved electron-accepting character within this molecular family, rather than as a strong acceptor in absolute terms. Its primary contribution lies in the modulation of the push–pull character, the intensification of the ICT state, and the extension of the absorption profile toward the visible and NIR regions, features that may be particularly advantageous in complementary light-harvesting strategies or multicomponent device architectures.

As a direct consequence, the energy gap ($E_{g}$) is drastically reduced in DTTQ-DPP-1, decreasing from approximately 2.70–2.71 eV in DTTQ-DPP to 0.82 eV in the gas phase and 0.86 eV in methanol, which favors the absorption of lower-energy photons and a better match with the solar spectrum, a key aspect for photovoltaic applications.

The ionization potential (I) and electron affinity (A) values follow these trends: DTTQ-DPP exhibits I = 4.31–4.58 eV and A = 1.60–1.87 eV, whereas DTTQ-DPP-1 shows a lower ionization potential (3.32–3.47 eV) and a markedly higher electron affinity (2.50–2.61 eV) within the studied series. This relative increase suggests an enhanced intrinsic electron-accepting character compared to DTTQ-DPP, although these values should not be interpreted as absolute benchmarks against established OPV acceptor materials.

The electronegativity (χ) ranges from 2.95 to 3.22 eV for DTTQ-DPP and from 2.91 to 3.04 eV for DTTQ-DPP-1, indicating a modest solvent effect. In contrast, the electrophilicity index (ω) increases significantly for DTTQ-DPP-1, reaching 10.32 eV (gas) and 10.77 eV (methanol), compared with 3.23 and 3.83 eV for DTTQ-DPP, indicating enhanced ground-state electron-accepting ability. The ICT character is therefore discussed based on the CDD maps, which directly evidence the excited-state charge redistribution.

**Figure 3:**
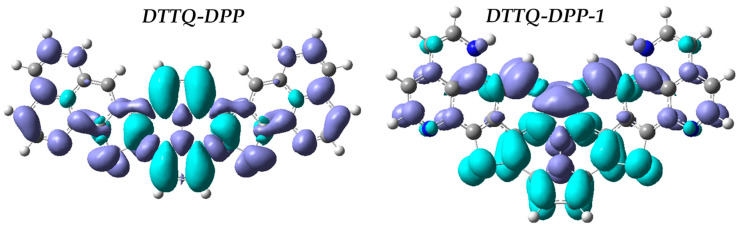
CDD for DTTQ-DPP and DTTQ-DPP-1. In the CDD maps, the cyan regions represent electron density accumulation, while the purple regions correspond to electron depletion (hole regions) upon excitation.

Figure 3 presents the charge density difference (CDD) maps of the dominant excited state for DTTQ-DPP and DTTQ-DPP-1. Both chromophores exhibit a typical push–pull charge redistribution upon photoexcitation, characterized by electron density depletion over the peripheral donor units and charge accumulation on the quinoxaline acceptor core. However, DTTQ-DPP-1 displays a more pronounced and spatially extended separation between hole and electron regions, indicating a more efficient intramolecular charge transfer process. This enhanced charge delocalization toward the acceptor center is consistent with the A–D–A molecular architecture and supports the ICT intensification induced by structural modification, in agreement with its improved optical and nonlinear optical properties.

The CDD was obtained by subtracting the ground-state electron density from the excited-state electron density corresponding to the S_0_→S_1_ transition. The calculations were performed at the CAM-B3LYP/6-311+G(d,p) level. The isosurfaces were visualized with a consistent isovalue for all systems to ensure proper comparison. In the CDD plots, cyan regions indicate electron density accumulation, while purple regions correspond to electron depletion (hole regions) upon excitation.

The dipole moment also increases upon moving from the gas phase to methanol, from 10.21 to 15.79 D for DTTQ-DPP and from 11.32 to 18.32 D for DTTQ-DPP-1, evidencing enhanced electronic polarization in polar media, which is favorable for exciton dissociation. Similarly, the polarizability increases significantly in solvent, reaching 111.81 × 10^−30^ esu for DTTQ-DPP and 227.88 × 10^−30^ esu for DTTQ-DPP-1, indicating a strong electronic response to external fields.

For the DTTQ-DPP chromophore in the gas phase, the comparison between wB97XD and B3LYP highlights intrinsic differences related to the exchange–correlation formalism of each functional. wB97XD is a range-separated hybrid functional that increases the fraction of exact exchange at long interelectronic distances and includes an explicit dispersion correction. As a result, it typically leads to a stronger stabilization of occupied orbitals and a larger HOMO–LUMO gap. This behavior is consistent with its improved performance in systems where nonlocal effects and charge-transfer contributions are relevant, since it mitigates the over-delocalization often associated with global hybrid functionals.

In contrast, B3LYP is a global hybrid functional with a fixed fraction of exact exchange, which generally produces a smaller HOMO–LUMO separation and, consequently, a reduced energy gap, as observed for DTTQ-DPP (2.70 eV). This feature is often associated with a “softer” description of π-conjugated systems and with higher predicted values of electron affinity and electrophilicity, favoring the interpretation of optoelectronic properties. Therefore, while wB97XD provides a more rigorous treatment of long-range exchange effects, B3LYP yields a more moderate estimation of the gap and reactivity, demonstrating how the choice of functional directly influences the electronic descriptors of the chromophore.

### 2.4. Thermodynamic Properties

Table 2 summarizes the main thermodynamic properties of the DTTQ-DPP and DTTQ-DPP-1 chromophores, calculated in the gas phase and in methanol at 298.15 K and 1 atm. In all cases, zero imaginary frequencies were obtained, confirming that the optimized geometries correspond to true minima on the potential energy surface and ensuring the structural stability of both systems in the environments considered.

The zero-point energy (ZPE) corrections show values of 264.10 kcal/mol (gas) and 264.50 kcal/mol (methanol) for DTTQ-DPP, whereas DTTQ-DPP-1 exhibits slightly lower values of 257.50 kcal/mol (gas) and 257.80 kcal/mol (methanol). This difference is associated with the different vibrational arrangement and the higher structural rigidity of DTTQ-DPP-1.

The thermal corrections to the energy and enthalpy follow a similar trend. For DTTQ-DPP, values of 282.10–282.30 kcal/mol (energy) and 282.70–283.00 kcal/mol (enthalpy) are obtained, while DTTQ-DPP-1 shows lower values of 275.30–275.50 kcal/mol and 275.90–276.10 kcal/mol, respectively. These results indicate that DTTQ-DPP-1 requires lower thermal contributions to maintain its energetic state.

The thermal correction to the Gibbs free energy is 227.50 kcal/mol (gas) and 227.90 kcal/mol (methanol) for DTTQ-DPP, and 221.90 kcal/mol (gas) and 222.30 kcal/mol (methanol) for DTTQ-DPP-1. The lower free energy values for DTTQ-DPP-1 suggest a different relative thermodynamic response between the two systems, particularly in a polar medium. Gibbs free energy differences mainly reflect vibrational and entropic contributions of isolated molecules and are used solely as comparative descriptors, without direct implications for device stability.

The total thermal energy (E) presents values consistent with the thermal corrections, reaching 282.07–282.33 kcal/mol for DTTQ-DPP and 275.30–275.51 kcal/mol for DTTQ-DPP-1. The constant-volume heat capacity (Cv) is around 120.76–120.27 cal·mol^−1^·K^−1^ for DTTQ-DPP and 122.31–121.93 cal·mol^−1^·K^−1^ for DTTQ-DPP-1, indicating a slightly higher thermal response for the latter, associated with its greater structural complexity and the larger number of accessible vibrational modes.

The entropy (S) shows values of 184.92–184.21 cal·mol^−1^·K^−1^ for DTTQ-DPP and 180.75–180.26 cal·mol^−1^·K^−1^ for DTTQ-DPP-1. The lower entropy observed for DTTQ-DPP-1 suggests a more ordered and rigid structure, consistent with its molecular design aimed at promoting coplanarity and extended conjugation.

**Figure 4:**
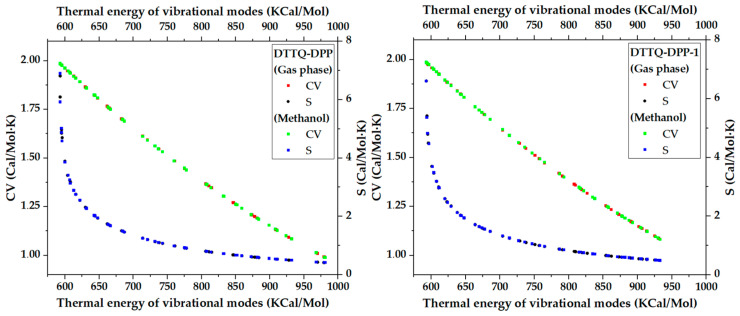
Comparison of vibrational heat capacity and entropy versus thermal energy in gas and methanol.

Figure 4 shows the evolution of the vibrational heat capacity (C_v_) and entropy (S) as a function of the thermal energy of the vibrational modes for the DTTQ-DPP and DTTQ-DPP-1 chromophores, comparing the results in the gas phase and in methanol. In both systems, C_v_ exhibits a progressive decrease as the thermal energy increases, a behavior consistent with the predominant contribution of low-energy vibrational modes to the overall thermal response, in accordance with general principles of statistical thermodynamics. Similarly, the entropy displays higher values at low energies and gradually decreases with increasing vibrational energy, reflecting the progressive reduction in the effective vibrational degrees of freedom.

When comparing the environments, the entropy values in methanol are slightly lower than those in the gas phase, suggesting increased structural rigidity and conformational restriction induced by the polar medium. Likewise, DTTQ-DPP-1 generally exhibits slightly lower entropy values and a thermal response in C_v_ comparable to that of DTTQ-DPP, which is consistent with a more ordered and rigid structure, in line with its molecular design aimed at promoting coplanarity and extended conjugation.

### 2.5. Optical Absorption and Fluorescence Properties

#### 2.5.1. Absorption

**Figure 5:**
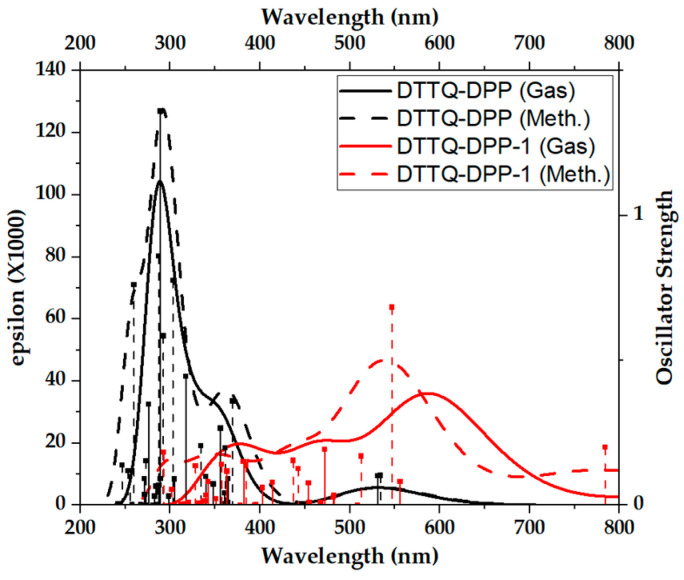
Absorption spectra of DTTQ-DPP in the gas phase and methanol.

Figure 5 presents the calculated absorption spectra (ε) of DTTQ-DPP in the gas phase (solid black line) and in methanol (dashed black line), together with the corresponding spectra of DTTQ-DPP-1 (red lines).

The figure reveals substantial differences in the optical response of both chromophores over a broad spectral range. In the case of DTTQ-DPP, the dominant absorption is mainly concentrated in the ultraviolet region, approximately between 260 and 320 nm, where high absorption coefficients are associated with π–π* electronic transitions. These transitions are primarily related to HOMO–LUMO excitations exhibiting significant spatial extension along the conjugated backbone; however, the net charge redistribution induced by excitation is limited, indicating a predominantly delocalized character rather than a pronounced intramolecular charge transfer (ICT). Consequently, its contribution to harvesting the visible portion of the solar spectrum is restricted.

In contrast, DTTQ-DPP-1 exhibits a significantly broader absorption profile, covering not only the ultraviolet region but also strongly extending into the visible range (400–650 nm), with additional contributions reaching the near-infrared (NIR). This spectral expansion is reflected in intense visible bands centered approximately between 500 and 600 nm, corresponding to clearly allowed electronic transitions. The oscillator strength analysis confirms that these bands arise from optically active excitations, with moderate f values for transitions around 531–534 nm, 470–480 nm, and 440–450 nm, indicating efficient interaction with electromagnetic radiation, although not necessarily exceeding that of chromophores possessing larger molar extinction coefficients.

The presence of low-energy transitions in DTTQ-DPP-1 extending beyond 700 nm suggests optical activity in the NIR region, associated with a reduced energy gap and excitations containing a stronger ICT component. Although these transitions exhibit lower oscillator strengths than the main visible bands, they broaden the spectral absorption range of the system. The visible transitions observed exclusively in methanol are not absent in the gas phase; rather, they display very small oscillator strengths in that environment, rendering their contribution to the convoluted spectrum negligible.

#### 2.5.2. Fluorescence

**Figure 6:**
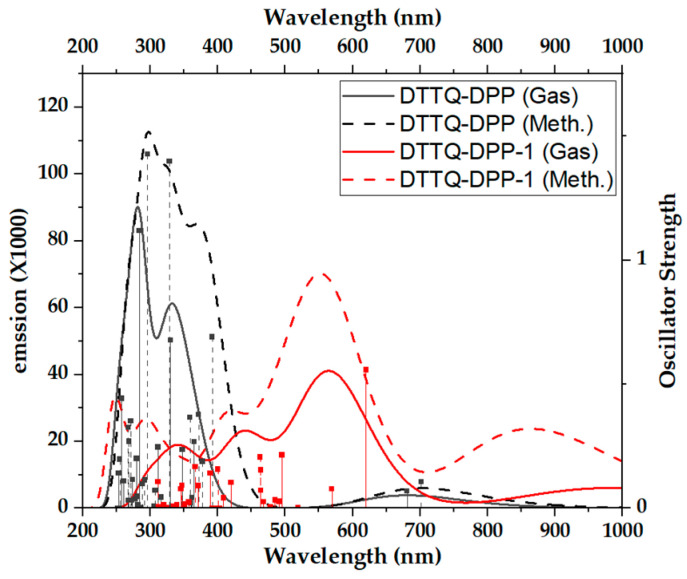
Fluorescence spectra of DTTQ-DPP in the gas phase and methanol.

From Figure 6, the calculated fluorescence spectra clearly reveal distinct emission behaviors between DTTQ-DPP and DTTQ-DPP-1, as well as a pronounced solvent effect. Since the geometries were optimized in the excited S_1_ state, the simulated fluorescence corresponds exclusively to the radiative S_1_→S_0_ transition in all cases. Therefore, the observed bands should be interpreted as broadened spectral profiles associated with this single transition, rather than as multiple independent electronic transitions.

In the case of DTTQ-DPP, the emission is primarily located in the ultraviolet–violet region, with maxima around 300–335 nm in both gas phase and methanol. This behavior is consistent with fluorescence dominated by π–π* excited states, involving moderate structural relaxation in the excited state, which results in a limited contribution within the visible region.

In contrast, DTTQ-DPP-1 exhibits intense and predominantly visible fluorescence, characterized by a broad band associated with the S_1_→S_0_ transition, centered in the green–yellow region. In the gas phase, the emission maximum is located around 560–570 nm, while in methanol, the emission becomes more intense and slightly shifts to shorter wavelengths, additionally displaying a spectral tail extending into the red and near-infrared regions. This spectral broadening and red-shifted emission are attributed to more pronounced structural relaxation in the S_1_ state and to a stronger intramolecular charge-transfer (ICT) character, favored by the push–pull architecture and by stabilization of the excited state in the polar medium.

Additional analysis of the charge density difference (CDD) maps for the S_1_ state supports this interpretation [49], revealing electron density redistribution from the peripheral donor units toward the acceptor core upon excitation, thereby confirming the ICT character assigned to DTTQ-DPP-1.

The proximity between the absorption and emission maxima in DTTQ-DPP reflects a small Stokes shift, consistent with limited structural relaxation in the excited state and a predominantly π–π* character. In contrast, the large Stokes shift observed for DTTQ-DPP-1 indicates a significant energetic separation between absorption and emission states, which may reduce optical reabsorption within the active layer. However, the actual impact of this feature on photovoltaic performance also depends on dynamic parameters such as excited-state lifetime and the competition between radiative and non-radiative processes, which were not explicitly evaluated in the present work.

### 2.6. Nonlinear Optical Properties

#### 2.6.1. First Hyperpolarizability

The results presented in Table 3 demonstrate a pronounced increase in both static and dynamic first hyperpolarizabilities for the Symmetric Thio-Bridge Quinoxaline Push–Pull organic chromophores, with DTTQ-DPP-1 exhibiting the most prominent nonlinear optical response. In the static regime, the $\beta \left(0;0,0\right)$ values of DTTQ-DPP remain relatively moderate, whereas a sharp enhancement is observed for DTTQ-DPP-1, reaching 975.80 × 10^−30^ esu in the gas phase and 1397.18 × 10^−30^ esu in methanol. This behavior becomes even more pronounced in the dynamic hyperpolarizability $\beta \left(-2\omega ;\omega ,\omega \right)$ at 1064 nm, which attains exceptionally high values of 1691.77 × 10^−30^ esu in gas and 3450.79 × 10^−30^ esu in methanol, indicating a strong nonlinear response under quasi-resonant conditions.

These large *β* values can be rationalized based on the optical properties discussed previously. The extended one-photon absorption toward the visible–NIR region, together with the intense two-photon absorption in the 800–1200 nm range, reveals the presence of low-lying excited states with pronounced intramolecular charge-transfer character. Such states promote large transition dipole moments and high electronic polarizability, which are key factors in amplifying the first-order nonlinear optical response. Additionally, the systematic enhancement of *β* in methanol highlights the role of the polar solvent in stabilizing charge-separated excited states, thereby reducing the energy denominators in response formalisms and significantly boosting the hyperpolarizability.

#### 2.6.2. Two-Photon Absorption

**Figure 7:**
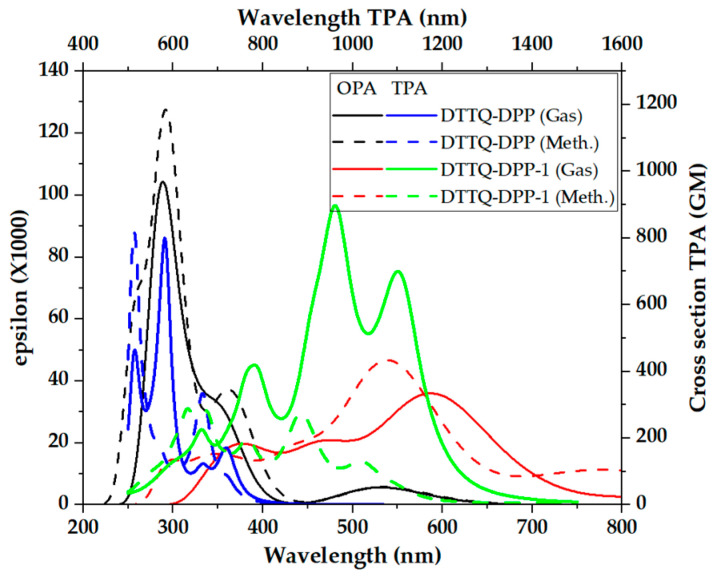
TPA and OPA spectra of Organic Chromophores Based on Symmetric Thio-Bridge Quinoxaline Push–Pull in gas phase and methanol.

As shown in Figure 7, chromophores based on Symmetric Thio-Bridge Quinoxaline Push–Pull exhibit clearly differentiated responses in one-photon absorption (OPA) and two-photon absorption (TPA) in both the gas phase and methanol. In the OPA regime, both systems display intense bands in the UV–Vis region, with a significant extension toward the visible and near-infrared regions, exceeding 800 nm, particularly for DTTQ-DPP-1 in methanol, which is highly favorable for photovoltaic applications. In contrast, the TPA spectra are predominantly located in the visible–NIR region, with maxima approximately spanning 800–1200 nm (TPA wavelength) and effective cross sections reaching values on the order of hundreds of Göppert–Mayer (GM) units, again being more pronounced for DTTQ-DPP-1 and in polar solvent.

Although TPA does not constitute an operative mechanism under standard solar illumination conditions (AM1.5G) due to the low incident photon density, the magnitude and spectral breadth of the TPA bands provide key intrinsic information regarding the electronic quality of the material. In particular, the high TPA values and their broad spectral distribution reflect strong intramolecular charge transfer, high electronic polarizability, extensive π-electron delocalization, and the presence of low-lying excited states with quasi-resonant coupling. These same features are responsible for the deep OPA observed in the NIR region and for a reduced effective energy gap, which are directly associated with efficient exciton separation and favorable charge transport. Consequently, while TPA does not directly enhance conventional photovoltaic performance, its analysis serves as a robust spectroscopic descriptor confirming the high optoelectronic potential of these push–pull chromophores for organic solar cell applications [50].

## 3. Discussion

The DTTQ-DPP and DTTQ-DPP-1 chromophores were designed as π-conjugated push–pull systems featuring a quinoxaline-based electron-accepting core and thio-conjugated bridges that ensure efficient intramolecular charge transfer. While DTTQ-DPP exhibits a typical organic donor behavior with a HOMO–LUMO gap of approximately 2.7 eV, DTTQ-DPP-1 displays a drastic reduction in the energy gap to 0.82–0.86 eV, accompanied by deep LUMO stabilization (−2.50 to −2.61 eV), a significant increase in the electrophilicity index (>10 eV), and enhanced polarizability. This combination of electronic descriptors accounts for its ability to absorb low-energy photons and extend the optical response beyond 800 nm, a spectral region that remains largely underexploited by conventional molecular chromophores. In addition, the lower Gibbs free energy and reduced entropy indicate a more rigid and stable molecular structure, which is favorable for its integration as an active material in next-generation organic solar cells.

The DTTQ-DPP-1 chromophore exhibits an excellent light absorption capability, positioning it as a highly promising material for optoelectronic and photovoltaic applications. Its push–pull architecture and extended π-conjugated system enable intense and broadband absorption, which is not limited to the ultraviolet region but extends significantly into the visible range (400–650 nm), with additional contributions toward the near-infrared (NIR). This spectral coverage matches well with the energy distribution of the solar spectrum, thereby maximizing the harvesting of useful photons. Moreover, the presence of multiple optically allowed electronic transitions, supported by appreciable oscillator-strength values, indicates efficient interaction with electromagnetic radiation. Overall, these features demonstrate that DTTQ-DPP-1 not only absorbs light effectively but does so in the most relevant spectral regions for photovoltaic conversion, promoting efficient exciton generation and potentially enhanced performance in organic solar cell devices.

While the presence of a low-energy intramolecular charge-transfer (ICT) state and a large Stokes shift in DTTQ-DPP-1 are generally associated with reduced optical reabsorption, the actual impact on photovoltaic device efficiency critically depends on the excited-state lifetime and on the competition between radiative and nonradiative deactivation processes, which were not explicitly evaluated in the present work.

The experimental absorption spectrum of the SD86 donor, reported in *Energy & Environmental Science*, shows intense absorption mainly in the visible region (450–650 nm), with a limited extension into the near-infrared that progressively decays before 800 nm [1]. This spectral coverage, together with careful morphological optimization and charge-transport management, enabled the achievement of photovoltaic efficiencies exceeding 18% in all-small-molecule devices [50].

In contrast, the theoretically estimated absorption spectrum of DTTQ-DPP-1 reveals a significantly more extended optical response, which not only effectively covers the 400–650 nm range but also clearly extends beyond 800 nm, penetrating directly into the deep near-infrared region. This feature represents a substantial difference compared to organic donors reported in the high-efficiency literature, where effective absorption rarely exceeds this spectral threshold [51,52]. The transitions responsible for this low-energy absorption are associated with excited states of pronounced intramolecular charge-transfer character, promoted by the push–pull architecture and the highly extended π-conjugated system of DTTQ-DPP-1.

The main novelty of DTTQ-DPP-1 therefore lies in its intrinsic ability to harvest photons in the >800 nm region, an area of the solar spectrum that remains partially underutilized in many commercial organic solar cells. This extension into the NIR suggests a potential increase in exciton generation from lower-energy photons, which could translate into an additional contribution to the short-circuit current, without the need to resort to ternary architectures or complex blend-engineering strategies.

Within the framework of this study, the computational results indicate that the incorporation of the push–pull architecture in DTTQ-DPP-1 leads to a significant reduction in the energy gap, an extended absorption coverage into the visible–NIR region, and enhanced intramolecular charge-transfer character. These features are relevant from a photovoltaic perspective, as they favor low-energy photon harvesting and charge separation. However, key device parameters such as effective VOC, active-layer morphology, and exciton dissociation dynamics would require further investigation for a quantitative assessment of actual device performance.

The results in Table 3 reveal contrasting hyperpolarizability behaviors for the two chromophores. DTTQ-DPP exhibits enhanced *β* values in a predominantly non-resonant regime, reflecting an intrinsic electronic response associated with its *push–pull* architecture. In contrast, the much larger *β* values obtained for DTTQ-DPP-1 at 1064 nm are strongly influenced by resonant electronic absorption and therefore do not represent a purely dispersive hyperpolarizability. From a photovoltaic perspective, this behavior is consistent with its extended absorption and reduced energy gap, which are advantageous for efficient solar light harvesting in organic solar cells.

Figure 7 shows a significant TPA response for the Symmetric Thio-Bridge Quinoxaline Push–Pull chromophores, with maxima between 800 and 1200 nm and cross sections on the order of hundreds of GM, particularly for DTTQ-DPP-1 in methanol. Although TPA is not operative under standard solar illumination, its high values confirm strong intramolecular charge transfer, high electronic polarizability, and low-lying excited states, acting as an intrinsic descriptor of the material’s optoelectronic quality.

## 4. Computational Methods

**Figure 8:**
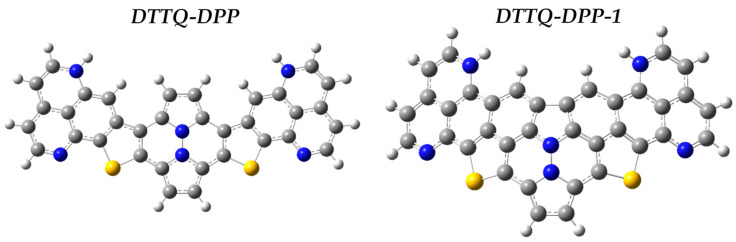
Molecular models in a ball-and-stick representation. Gray spheres represent carbon atoms, white spheres correspond to hydrogen, blue spheres indicate nitrogen, and yellow spheres represent sulfur, allowing a clear identification of the atomic composition and connectivity of each molecule.

The molecules shown in Figure 8 correspond to conjugated organic systems with a symmetric architecture, composed of multiple interconnected heteroaromatic rings (phenyl and pyridine units), which favor extensive electronic delocalization along the molecular backbone. The presence of nitrogen atoms integrated into the heteroaromatic rings introduces electron-accepting sites, whereas sulfur atoms act as thioether or thio-heterocyclic bridges that enhance π-conjugation and the polarizability of the system. This structural combination gives rise to push–pull chromophores (DTTQ-DPP and DTTQ-DPP-1), in which intramolecular charge transfer from electronically rich regions toward more electronegative regions is facilitated. The nearly planar arrangement of the aromatic fragments promotes π-orbital overlap, favoring relevant optical and electronic properties.

The compounds DTTQ-DPP (C_34_H_16_N_6_S_2_) and DTTQ-DPP-1(C_36_H_12_N_8_S_2_) were initially designed using the Avogadro software version 1.102.0 [53], which allows the construction and visualization of molecular structures through the explicit definition of atomic coordinates, atom types, and chemical bonds. Subsequently, the molecular geometries were optimized in the ground state using the Gaussian16 program, applying the standard convergence criteria of the software [54]. The optimizations were carried out using time-independent Density Functional Theory (DFT), with the hybrid B3LYP functional and the 6-31G+(d,p) basis set, which incorporates diffuse and polarization functions necessary to adequately describe conjugated organic systems and their electronic properties [55,56].

The solvent effects (methanol) were included using the implicit solvation model CPCM (Conductor-like Polarizable Continuum Model), in which the medium is represented as a polarizable dielectric continuum characterized by its dielectric constant [57,58]. This approach enables an efficient evaluation of the electrostatic influence of the environment on the energetic and electronic properties of the solute without explicitly including solvent molecules. In this work, methanol was employed as a polar reference medium to analyze the environmental sensitivity of the chromophores and the relative stabilization of states with intramolecular charge-transfer (ICT) character. Therefore, the results obtained in methanol should be interpreted as an estimation of dielectric screening effects under a higher-polarity limit, rather than as a direct simulation of the actual processing conditions in OSC devices, where solvents of lower polarity are typically used.

From the energies of the frontier molecular orbitals HOMO and LUMO, and employing Koopmans’ approximation, several electronic descriptors and intrinsic chemical reactivity parameters were calculated, such as ionization potential, electron affinity, electronegativity, and chemical hardness [59]. Additionally, the checkpoint files (.chk) generated in Gaussian16 were used to visualize the HOMO–LUMO frontier orbitals and electrostatic potential maps, enabling the analysis of electron density distribution and the localization of electron-donor and electron-acceptor regions.

All calculations were carried out within the framework of Density Functional Theory (DFT) using the B3LYP and wB97XD exchange–correlation functionals to assess the influence of the functional choice on the electronic properties of DTTQ-DPP in the gas phase. B3LYP, a global hybrid functional, was employed as a conventional reference, while wB97XD, a range-separated hybrid functional with long-range exact exchange and dispersion correction, was used to account for nonlocal electronic effects [55]. This comparative approach enables evaluation of the sensitivity of frontier orbital energies and related descriptors to the exchange–correlation treatment.

In order to verify that the optimized structures correspond to true minima on the potential energy surface, vibrational frequency calculations were performed at the same level of theory, confirming the absence of imaginary frequencies [60]. The absence of imaginary frequencies is reported solely as a computational criterion to validate the optimized geometries. From these calculations, thermodynamic parameters such as temperature (298.15 K), pressure (1 atm), zero-point energy correction, thermal corrections to energy, enthalpy, and Gibbs free energy, as well as thermal energy, constant-volume heat capacity (Cv), and entropy (S), were obtained. These results allowed a comparative analysis of vibrational heat capacity and vibrational entropy as a function of thermal energy, both in the gas phase and in methanol.

UV–Vis absorption spectra were calculated using Time-Dependent Density Functional Theory (TD-DFT) [61], considering 20 singlet excited states, employing the range-separated CAM-B3LYP functional together with the 6-311+G(d,p) basis set [30]. The simulated spectra were obtained by applying a Gaussian convolution with a full width at half maximum (FWHM) of 0.24 eV. The Charge Density Difference (CDD) maps were generated using the Multiwfn 3.8 program based on the TD-DFT results.

The molar absorptivity $\varepsilon \left(\omega \right)$ in the UV–Vis spectrum was calculated from the superposition of Lorentzian functions according to Equation (1) [38].

(1) $$\varepsilon \left(\omega \right)=\frac{N_{A}e^{2}\ln10}{4\pi m_{e}c^{2}\varepsilon_{0}}\sum_{i}f_{i}\frac{\Gamma /2\;}{{\left(\omega -\omega_{0}\right)}^{2}+{\left(\frac{\Gamma }{2}\right)}^{2}},$$

In this expression, each Lorentzian peak presents an area proportional to the oscillator strength $f_{i}$ and a linewidth Γ, scaled by fundamental constants. This formulation allows a realistic spectral representation that reflects both the probability and energy of each electronic transition. In contrast, the calculation of emission spectra corresponds to a downward electronic transition process, which is computationally more demanding.

Emission spectra were calculated by previously optimizing the molecular geometry in the first singlet excited state, ensuring an adequate description of the radiative relaxation process. Subsequently, TD-DFT calculations were performed for 20 singlet states at the same CAM-B3LYP/6-311+G(d,p) level, applying a Gaussian convolution with an FWHM of 0.24 eV.

The first hyperpolarizability (β) was determined from the components of the hyperpolarizability tensor obtained at the same level of theory as the optical calculations, considering a wavelength of 1063 nm. The effective values were calculated using the Kamada method. In isotropic media, the orientational average corresponding to hyper-Rayleigh scattering (HRS) was obtained according to Equation (2) [62].

(2) $$\langle \beta_{HRS}^{2}\rangle =\frac{1}{30}\sum_{i}\sum_{j,k}\beta_{ijk}^{2}+\frac{1}{15}\sum_{i\neq j}\beta_{iij}\beta_{jji},$$

From this expression, the effective hyperpolarizability was obtained using Equation (3).

(3) $$\beta_{HRS}=\sqrt{\langle \beta_{HRS}^{2}\rangle },$$

Two-photon absorption (TPA) spectra were simulated using the sum-over-states (SOS) method, employing the TD-DFT results as input. The spectral simulation was performed by applying a Gaussian convolution with an FWHM of 0.24 eV, yielding realistic spectral profiles comparable to experimental data reported in the literature.

The orientational average of the squared two-photon transition matrix element was obtained from the transition dipole moments, calculated from the excitation energy values ΔE and oscillator strengths f extracted from Gaussian output files (out and fch), according to Equation (4).

(4) $$\left|\mu_{fg}\right|=\sqrt{\frac{2f}{3\Delta E}},$$

Subsequently, the orientational average was obtained by summing the contributions of all simulated states, as shown in Equation (5).

(5) $$\langle {\left|M_{fg}^{2}\right|}^{2}\rangle =\sum_{i}{\left|\mu_{fg}\right|}_{i},$$

Finally, the two-photon absorption cross section was calculated using Equation (6).

(6) $$\sigma^{2}\left(\omega \right)=\frac{8\pi^{3}\omega^{2}}{c^{2}n^{2}}\langle {\left|M_{fg}^{2}\right|}^{2}\rangle g\left(2\omega \right),$$

In this expression, $\sigma^{2}\left(\omega \right)$, expressed in Göppert–Mayer (GM) units, depends on the frequency of the incident radiation, the refractive index of the medium, and the broadening function $g\left(2\omega \right)$, corresponding to the simultaneous absorption of two photons.

## 5. Conclusions

The objective of designing and theoretically characterizing push–pull organic chromophores based on Symmetric Thio-Bridge Quinoxaline Push–Pull for organic solar cell applications was successfully achieved by establishing a coherent relationship between molecular structure, intramolecular charge transfer, and optoelectronic performance. The central finding is that the structural modulation leading to DTTQ-DPP-1 markedly enhances its electron-accepting character and electronic polarization, as reflected in LUMO stabilization, an increased electrophilicity index, higher dipole moment, and enhanced polarizability, together with a reduced energy gap that supports absorption extended into the near-infrared region and a more favorable optical interaction with the solar spectrum compared to DTTQ-DPP.

In comparison with widely used organic donors in solar cells, such as P3HT, PTB7, PM6, or small-molecule systems reported with efficiencies exceeding 15–18% [45,46,50,63], DTTQ-DPP-1 exhibits an optical response shifted toward longer wavelengths and a significantly smaller band gap, indicating a distinct electronic regime oriented toward harvesting lower-energy photons beyond the visible region. Furthermore, the absence of imaginary frequencies and the observed thermodynamic trends support the structural viability of both chromophores, with DTTQ-DPP-1 standing out for its relatively higher rigidity, consistent with its conjugated molecular design.

As a strength, this work systematically integrates electronic descriptors, intrinsic chemical reactivity, thermodynamic analysis, absorption and emission properties, and nonlinear optical parameters using DFT and TD-DFT, providing molecular design criteria that are comparable to those of benchmark photovoltaic materials. Among the limitations, the results rely on implicit solvation and do not incorporate explicit effects of aggregation, thin-film morphology, interfaces, or excitonic dynamics, which are critical determinants of real device efficiency. Additionally, the dynamic hyperpolarizability calculated at 1064 nm may be influenced by quasi-resonant contributions and therefore should not be interpreted as a purely dispersive response.

## Figures and Tables

**Table 1:** Electronic descriptors and intrinsic chemical reactivity.

Property	DTTQ-DPP		DTTQ-DPP-1		DTTQ-DPP
	b3lyp/6-31g+(d,p)				wb97xd/dgdzvp2
	(Gas)	(Methanol)	(Gas)	(Methanol)	(Gas)
Energía total (eV)	−66,129.13	−66,129.79	−68,167.68	−68,168.36	−66,120.44
HOMO (eV)	−4.31	−4.58	−3.32	−3.47	−6.34
LUMO (eV)	−1.60	−1.87	−2.50	−2.61	−0.22
Energy gap $E_{g}$ (eV)	2.70	2.71	0.82	0.86	6.12
Ionization potential I (eV)	4.31	4.58	3.32	3.47	6.34
Electron affinity A (eV)	1.60	1.87	2.50	2.61	0.22
Electronegativity χ (eV)	2.95	3.22	2.91	3.04	3.28
Electrophilicity index ω (eV)	3.23	3.83	10.32	10.77	1.76
Dipole moment (Debye)	10.21	15.79	11.32	18.32	10.29
$\mathrm{Polarizability}\;(\times 10^{-30}$ esu)	80.10	111.81	115.10	227.88	---

The data were obtained from .out files by the authors.

**Table 2:** Thermodynamic properties.

Property	Units	DTTQ-DPP		DTTQ-DPP-1	
		(Gas)	(Methanol)	(Gas)	(Methanol)
Imaginary frequencies	—	0	0	0	0
Temperature	K	298.15	298.15	298.15	298.15
Pressure	atm	1.00	1.00	1.00	1.00
Zero-point energy correction	kcal/mol	264.10	264.50	257.50	257.80
Thermal correction to energy	kcal/mol	282.10	282.30	275.30	275.50
Thermal correction to enthalpy	kcal/mol	282.70	283.00	275.90	276.10
Thermal correction to Gibbs free energy	kcal/mol	227.50	227.90	221.90	222.30
Thermal energy (E)	kcal/mol	282.07	282.33	275.30	275.51
Heat capacity (Cv)	cal·mol^−1^·K^−1^	120.76	120.27	122.31	121.93
Entropy (S)	cal·mol^−1^·K^−1^	184.92	184.21	180.75	180.26

The data were obtained from .out files by the authors.

**Table 3:** Static $\beta \left(0;0,0\right)$ and dynamic $\beta \left(-2\omega ;\omega ,\omega \right)$ first hyperpolarizabilities at 1064 nm of Organic Chromophores Based on Symmetric Thio-Bridge Quinoxaline Push–Pull.

Chromophores		$\beta \left(0;0,0\right)$ $\times 10^{-30}\;esu$	$\beta \left(-2\omega ;\omega ,\omega \right)$ $\times 10^{-30}\;esu$
DTTQ-DPP	Gas	15.77	38.81
	Methanol	22.58	58.51
DTTQ-DPP-1	Gas	975.80	1691.77
	Methanol	303.85	3450.79

The data were obtained from .out files by the authors.

## Data Availability

The data supporting the findings of this study, including optimized structures, electronic properties, and nonlinear optical responses, were obtained through quantum chemical calculations using Gaussian software. These data are available from the corresponding author upon reasonable request.

## Abbreviations

The following abbreviations are used in this manuscript:

DFT	Density Functional Theory
TD-DFT	Time-Dependent Density Functional Theory
B3LYP	Becke, 3-parameter, Lee–Yang–Parr functional
CAM-B3LYP	Coulomb-Attenuating Method–B3LYP
Homo	Highest Occupied Molecular Orbital
Lumo	Lowest Unoccupied Molecular Orbital
FWHM	Full Width at Half Maximum
SOS	Sum-over-states
CPCM	Conductor-like Polarizable Continuum Model

## References

[1] Gao Y., Xu L.Y., Chen X., Xiao B., Gao W., Xia J., Sun R., Min J. (2025). Highly efficient all-small-molecule organic solar cells with excellent operational stability and blend-thickness tolerance. Energy Environ. Sci..

[2] Li M., Chen G., Lan A., Chung S., Que M., Cho Y., Huang B. (2025). Constructing high-performance solar cells by incorporating an A1–A2-type polymer donor as a guest material. Molecules.

[3] Aziza A.A., Mohamed E., Zaitsev I., Vladislav K. (2026). HDTMS-, polybutadiene-, and benzotriazole-modified polylactic-based resin for solar cells encapsulation with exceptional environmental stability of MAPI perovskite films. Molecules.

[4] Xu Y., Yao H., Ma L., Hong L., Li J., Liao Q., Zu Y., Wang J., Gao M., Ye L. (2020). Tuning the hybridization of local exciton and charge-transfer states in highly efficient organic photovoltaic cells. Angew. Chem. Int. Ed..

[5] Veldman D., Meskers S.C.J., Janssen R.A.J. (2009). The energy of charge-transfer states in electron donor–acceptor blends: Insight into the energy losses in organic solar cells. Adv. Funct. Mater..

[6] Terenziani F., Katan C., Badaeva E., Tretiak S., Blanchard-Desce M. (2008). Enhanced two-photon absorption of organic chromophores: Theoretical and experimental assessments. Adv. Mater..

[7] Yang Y., Wang H., Liu F., Yang D., Bo S., Qiu L., Zhen Z., Liu X. (2015). The synthesis of new double-donor chromophores with excellent electro-optic activity by introducing modified bridges. Phys. Chem. Chem. Phys..

[8] Patil Y., Butenschön H., Misra R. (2023). Tetracyanobutadiene bridged push–pull chromophores: Development of new generation optoelectronic materials. Chem. Rec..

[9] Waqas M., Khadka D.B., Khan A.H.H., Wang Y.C. (2025). Fullerene-driven photocarrier processes in perovskite solar cells: Recent advances. Nanoscale.

[10] Zhang Y., Zhang S., Dai W., Yang S. (2025). Versatile roles of functionalized fullerenes in perovskite solar cells. J. Mater. Chem. A.

[11] Gaponenko I.N., Ageev S.V., Iurev G.O., Shemchuk O.S., Meshcheriakov A.A., Petrov A.V., Solovtsova I.L., Vasina L.V., Tennikova T.B., Murin I.V. (2020). Biological evaluation and molecular dynamics simulation of water-soluble fullerene derivative C_60_[C(COOH)_2_]_3_. Toxicol. Vitr..

[12] Wu X., Yang X., Chang B., Sun R., Min J. (2025). Material insights and challenges for organic photovoltaics based on non-fullerene acceptors. Joule.

[13] Yaqoob U., Ayub A.R., Rafiq S., Khalid M., El-Badry Y.A., El-Bahy Z.M., Iqbal J. (2021). Structural, optical and photovoltaic properties of unfused non-fullerene acceptors for efficient solution processable organic solar cell: A DFT approach. J. Mol. Liq..

[14] Faisal R.M., De Silva R., Nalin De Silva K.M., Lanka S. (2022). Density functional theory simulations on fullerene/polymer blends for organic photovoltaic systems. Int. J. Adv. Res. Ideas Innov. Technol..

[15] Thanmayalaxmi D., Suvitha A., Sakthivel P., Alharbi N., Florence S., Trilaksana H., El-Mansy M.A.M. (2024). Quantum Computational and Spectroscopic Investigation (UV), MEP, HOMO–LUMO, Pharmacokinetic Studies of Meloxicam: A DFT Study.

[16] Elangovan N., Sowrirajan S., Arumugam N., Rajeswari B., Mathew S., Priya C.G., Venkatraman B.R., Mahalingam S.M. (2024). Theoretical investigation on solvent effects in molecular structure (TD-DFT, MEP, HOMO–LUMO), topological analysis and molecular docking studies. Polycycl. Aromat. Compd..

[17] Tian H., Yang X., Pan J., Chen R., Liu M., Zhang Q., Hagfeldt A., Sun L. (2008). A triphenylamine dye model for the study of intramolecular energy transfer and charge transfer in dye-sensitized solar cells. Adv. Funct. Mater..

[18] Jia L., Wu Q., Yang T., Xie B., Sheng J., Xie W., Shi J. (2025). BN-doped polycyclic aromatic hydrocarbons and their applications in optoelectronics. Molecules.

[19] Daher A., Choudhari M., Roland T., De Waele V., Daniele S. (2025). Zinc β-diketonates with donor–acceptor ligands: Synthesis and comprehensive structural, thermal, and photophysical characterization. Molecules.

[20] Orfanos I., Aloukos P., Kaloudi-Chantzea A., Pistolis G., Couris S. (2025). Efficient tuning of the third-order nonlinear optical properties of functionalized boron-dipyrromethene dyes. Nanomaterials.

[21] Nicolas P., Abdallah S., Chen D., Rizzi G., Jeannin O., Clays K., Bellec N., Bilgin-Eran B., Akdas-Kiliç H., Malval J.P. (2025). From chains to chromophores: Tailored thermal and linear/nonlinear optical features of asymmetric pyrimidine–coumarin systems. Molecules.

[22] Yokoyama M., Kishi R., Kitagawa Y. (2024). Theoretical study on one- and two-photon absorption properties of π-stacked multimer models of phenalenyl radicals. Chemistry.

[23] Grandhi G.K., Koutsourakis G., Blakesley J.C., De Rossi F., Brunetti F., Öz S., Sinicropi A., Parisi M.L., Brown T.M., Carnie M.J. (2025). Promises and challenges of indoor photovoltaics. Nat. Rev. Clean Technol..

[24] Mujtaba Babar M., Anvari B., Ersoy G., Henary M. (2025). Roadmap for designing donor–π–acceptor fluorophores in UV–Vis and NIR regions: Synthesis, optical properties and applications. Biomolecules.

[25] Bravo N.F., Cifuentes C., Ríos M.C., Pérez L.J., Portilla J. (2025). Synthesis and photophysical properties of conjugated fluorophores with N-heteroaromatic rings. Asian J. Org. Chem..

[26] Coropceanu V., Chen X.K., Wang T., Zheng Z., Brédas J.L. (2019). Charge-transfer electronic states in organic solar cells. Nat. Rev. Mater..

[27] Wang C.H., Adachi Y., Ohshita J. (2024). Synthesis of unsymmetrically condensed benzo- and thienotriazologermoles. Molecules.

[28] Maadh F.N., Abdulmalek E., Ismail M.F., Ahmad S.A.A., Abdulkreem-Alsultan G. (2024). Recent progress in benzimidazole and quinoxaline dyes based on dye-sensitized solar cells: A comprehensive overview. High Energy Chem..

[29] Long Y., Chen K., Li C., Wang W., Bian J., Li Y., Liu S., Chi Z., Xu J., Zhang Y. (2023). Molecular design strategy for through-space charge transfer blue polyimides with rigid non-conjugated backbone and the role of alicyclic imide linker. Chem. Eng. J..

[30] Manzhos S., Rivera E., Avila O., Fonseca R. (2026). Functionalized benzoxazole–pyrimidine derivatives for deep bioimaging: A DFT study of molecular architecture and one- and two-photon absorption. Physchem.

[31] Shakoor A., Jan F., Rahman S., Ali M., Ibrahim M., Khan H., Alam A., Khan A., Ali A., Al-Olayan E. (2025). Synthesis, urease inhibitory activity, molecular docking, dynamics, MMGBSA and DFT studies of hydrazone-Schiff bases bearing benzimidazole scaffold. Chem. Biodivers..

[32] Behjatmanesh-Ardakani R., Imanov H.A. (2025). DFT study on the mechanism of benzimidazole synthesis from phenylenediamine and formic acid: Activation energies and transition states’ locations. Chem. Rev. Lett..

[33] Samuilov Y.D., Samuilov A.Y. (2024). Reaction of N-methylformamide with dimethyl carbonate: A DFT study. Theor. Chem. Acc..

[34] Mahmoudi C., Chouk R., Baatout K., Jaballah N.S., Khalfaoui M., Majdoub M. (2022). Synthesis, characterization and DFT study of new anthracene-based semiconducting polyethers for OLED application. J. Mol. Struct..

[35] Zhou Z., Song J., Xie Y., Ma Y., Hu H., Li H., Zhang L., Lawrie C.H. (2025). DFT calculation for organic semiconductor-based gas sensors: Sensing mechanism, dynamic response and sensing materials. Chin. Chem. Lett..

[36] Kalavathi A., Saravanakumar P., Satheeshkumar K., Vennila K.N., Elango K.P. (2023). Spectral and DFT/TD-DFT studies on turn-on fluorescent detection of Al(III) by a quinolin-8-ol-based Schiff base and its bioimaging. J. Mol. Struct..

[37] Sakr M.A.S., Sherbiny F.F., El-Etrawy A.A.S. (2022). Hydrazone-based materials: DFT, TD-DFT, NBO analysis, Fukui function, MESP analysis and solar cell applications. J. Fluoresc..

[38] Rivera E., Ceballo R., Neira O., Avila O., Fonseca R. (2025). DFT study of functionalized benzoxazole-based D–π–A architectures: Influence of ionic fragments on optical properties and their potential in OLED and solar cell devices. Molecules.

[39] Krasley A.T., Li E., Galeana J.M., Bulumulla C., Beyene A.G., Demirer G.S. (2024). Carbon nanomaterial fluorescent probes and their biological applications. Chem. Rev..

[40] Murugan P., Liu S.Y. (2025). Innovative approaches to the molecular design, synthesis and functionalization of conjugated organic polymer photocatalysts for sustainable hydrogen production. J. Mater. Chem. A.

[41] Le P.G., Kim D., Chung J.P., Cho S. (2025). Peroxidase-mimicking nanozymes of nitrogen heteroatom-containing graphene oxide for biomedical applications. Biosensors.

[42] Chen X., Feng P., Zheng Y., Li H., Zhang Y., Shen Y., Yan Y., Liu M., Ye L. (2025). Emerging nitrogen- and sulfur-codoped carbon materials for electrochemical energy storage and conversion. Small.

[43] Haque A., Alenezi K.M., Khan M.S., Wong W.Y., Raithby P.R. (2023). Non-covalent interactions in π-conjugated functional materials: Advances and perspectives. Chem. Soc. Rev..

[44] Masood I., Singh M.P., Amir M. (2026). P3HT:PCBM-based organic solar cells: Through strategic ETL and HTL material selection. Eng. Res. Express.

[45] Alahmadi A.N.M. (2022). Design of an efficient PTB7:PC70BM-based polymer solar cell for 8% efficiency. Polymers.

[46] Sun C., Li S., Kuvondikov V., Nematov S., Ye L. (2025). Unlocking intrinsic stretchability in PM6-based materials for next-generation solar cells: Challenges and innovations. Sci. China Mater..

[47] Zhang C.R., Yu H.Y., Zhang M.L., Liu X.M., Chen Y.H., Liu Z.J., Wu Y.Z., Chen H.S. (2023). Modulating the organic photovoltaic properties of non-fullerene acceptors by molecular modification based on Y6: A theoretical study. Phys. Chem. Chem. Phys..

[48] Park J.S., Kim G.U., Lee S., Lee J.W., Li S., Lee J.Y., Kim B.J. (2022). Material design and device fabrication strategies for stretchable organic solar cells. Adv. Mater..

[49] Mufeed M., Sirohi A., Singh J. Physisorption interaction of nucleobases on ZrGeTe4 using density functional theory study for biomolecule sensing. Proceedings of the 2024 8th IEEE Electron Devices Technology & Manufacturing Conference (EDTM).

[50] Peng W., Lin Y., Jeong S.Y., Genene Z., Magomedov A., Woo H.Y., Chen C., Wahyudi W., Tao Q., Deng J. (2022). Over 18% ternary polymer solar cells enabled by a terpolymer as the third component. Nano Energy.

[51] Zhang H., Lu B., Liu D. (2022). Methods for increasing the conversion efficiency of solar cells. J. Phys. Conf. Ser..

[52] Gu K., Zhong H. (2023). A general methodology to measure the light-to-heat conversion efficiency of solid materials. Light Sci. Appl..

[53] Snyder H.D., Kucukkal T.G. (2021). Computational chemistry activities with Avogadro and ORCA. J. Chem. Educ..

[54] Deringer V.L., Bartók A.P., Bernstein N., Wilkins D.M., Ceriotti M., Csányi G. (2021). Gaussian process regression for materials and molecules. Chem. Rev..

[55] Nakata M., Maeda T. (2023). PubChemQC B3LYP/6-31G*//PM6 data set: The electronic structures of 86 million molecules using B3LYP/6-31G* calculations. J. Chem. Inf. Model..

[56] Eglitis R.I., Jia R. (2023). Review of systematic tendencies in (001), (011) and (111) surfaces using B3PW as well as B3LYP computations of perovskites. Materials.

[57] Ringe S., Hörmann N.G., Oberhofer H., Reuter K. (2021). Implicit solvation methods for catalysis at electrified interfaces. Chem. Rev..

[58] Severoglu Y.B., Yuksel B., Sucu C., Aral N., Uversky V.N., Coskuner-Weber O. (2025). Implicit solvent models and their applications in biophysics. Biomolecules.

[59] Khaldi M.K., Al-Dhaifallah M., Aljamaan I., Al-Sunni F.M., Taha O., Alharbi A. (2025). From block-oriented models to the Koopman operator: A comprehensive review on data-driven chemical reactor modeling. Mathematics.

[60] Kraus J., Brehm S., Himcinschi C., Kortus J. (2024). Structural and Thermodynamic Properties of Filter Materials: A Raman and DFT Investigation.

[61] Idrissi S., Labrim H., Bahmad L., Benyoussef A. (2021). DFT and TDDFT studies of the new inorganic perovskite CsPbI3 for solar cell applications. Chem. Phys. Lett..

[62] Kamada K. (2025). Experimental methods and nonlinear optical properties of open-shell molecular species. Chemistry.

[63] Balraj B., Raja M., Prabhu T.G.V., Balaji M., Chandrasekaran J., Nagarajan S.K., Bharathi M., Lee S., Sivakumar C. (2025). A critical review on decade progress and future prospects of P3HT:PCBM bulk heterojunction solar cells. Sol. Energy.

